# Data-Driven Exploration of National Health Service Talking Therapies Care Pathways Using Process Mining: Retrospective Cohort Study

**DOI:** 10.2196/53894

**Published:** 2024-05-21

**Authors:** Elizabeth Yardley, Alice Davis, Chris Eldridge, Christos Vasilakis

**Affiliations:** 1 School of Management, University of Bath Bath United Kingdom; 2 Mayden Bath United Kingdom

**Keywords:** electronic health record, EHR, electronic health records, EHRs, health record, data science, secondary data analysis, mental health services, mental health, health information system, HIS, information system, information systems, process mining, flow, flows, path, pathway, pathways, delivery, visualization

## Abstract

**Background:**

The National Health Service (NHS) Talking Therapies program treats people with common mental health problems in England according to “stepped care,” in which lower-intensity interventions are offered in the first instance, where clinically appropriate. Limited resources and pressure to achieve service standards mean that program providers are exploring all opportunities to evaluate and improve the flow of patients through their service. Existing research has found variation in clinical performance and stepped care implementation across sites and has identified associations between service delivery and patient outcomes. Process mining offers a data-driven approach to analyzing and evaluating health care processes and systems, enabling comparison of presumed models of service delivery and their actual implementation in practice. The value and utility of applying process mining to NHS Talking Therapies data for the analysis of care pathways have not been studied.

**Objective:**

A better understanding of systems of service delivery will support improvements and planned program expansion. Therefore, this study aims to demonstrate the value and utility of applying process mining to NHS Talking Therapies care pathways using electronic health records.

**Methods:**

Routine collection of a wide variety of data regarding activity and patient outcomes underpins the Talking Therapies program. In our study, anonymized individual patient referral records from two sites over a 2-year period were analyzed using process mining to visualize the care pathway process by mapping the care pathway and identifying common pathway routes.

**Results:**

Process mining enabled the identification and visualization of patient flows directly from routinely collected data. These visualizations illustrated waiting periods and identified potential bottlenecks, such as the wait for higher-intensity cognitive behavioral therapy (CBT) at site 1. Furthermore, we observed that patients discharged from treatment waiting lists appeared to experience longer wait durations than those who started treatment. Process mining allowed analysis of treatment pathways, showing that patients commonly experienced treatment routes that involved either low- or high-intensity interventions alone. Of the most common routes, >5 times as many patients experienced direct access to high-intensity treatment rather than stepped care. Overall, 3.32% (site 1: 1507/45,401) and 4.19% (site 2: 527/12,590) of all patients experienced stepped care.

**Conclusions:**

Our findings demonstrate how process mining can be applied to Talking Therapies care pathways to evaluate pathway performance, explore relationships among performance issues, and highlight systemic issues, such as stepped care being relatively uncommon within a stepped care system. Integration of process mining capability into routine monitoring will enable NHS Talking Therapies service stakeholders to explore such issues from a process perspective. These insights will provide value to services by identifying areas for service improvement, providing evidence for capacity planning decisions, and facilitating better quality analysis into how health systems can affect patient outcomes.

## Introduction

### The National Health Service Talking Therapies Program

Common mental health disorders such as anxiety and depression are among the top causes of disability in England [1], affecting 1 in 6 adults [2], and their prevalence is increasing [2,3]. This increasing health burden is reflected in the growing number of referrals received by the National Health Service (NHS) Talking Therapies program (formerly Improving Access to Psychological Therapies [IAPT]) since its inception in 2008 [4]. Each year, >1 million patients access Talking Therapies through the program [5], and like all parts of the NHS in England, the program is constrained by limited resources while facing additional pressure to achieve service standards for recovery rates and waiting times. On the basis of 2021 to 2022 access rates [5], the volume of people receiving treatment through the program will need to increase by 50% by 2024 to meet the targets set out in the NHS Long Term Plan [6]. Therefore, providers are looking at all opportunities to increase capacity and productivity to better understand and improve the flow of patients through care systems and to evaluate the performance of their services.

The Talking Therapies treatment model is based on the principle of stepped care, which is the approach recommended by the National Institute for Health and Care Excellence (NICE) [7]. The stepped care approach maintains that those with milder conditions should be treated initially with lower-intensity interventions, and these individuals might then be “stepped up” to higher-intensity interventions if clinically appropriate [7]. The stepped care approach has been shown to be associated with improved patient outcomes [8,9].

As detailed in Table 1, the first step in the stepped care model represents the presentation of a common mental health problem. This initial presentation is usually followed by a referral to an NHS Talking Therapies service, which generally offers step 2 and 3 interventions, while more specialist services offer higher-intensity interventions at steps beyond this.

While each therapy service will follow the overall model of care prescribed by the IAPT model [11], each service has a uniquely configured care pathway, which represents the plan for the implementation of clinical guidance. Studies have shown that there is variation in how the principle of stepped care is implemented [12,13], and clinical performance has also been shown to vary across Talking Therapies program sites [9,14]. Despite this variation in implementation and performance, methods of implementation and systems of treatment delivery are relatively understudied in comparison to the psychological therapies offered [12,13].

Studies have demonstrated the relevance of implementation methods to the program’s performance by identifying features of service delivery that are predictive of clinical outcomes and patient engagement. For instance, longer waiting times have been associated with patient disengagement with IAPT therapy services [15,16] and worse recovery outcomes [9]. More treatment sessions, a larger service size, and a greater proportion of therapy sessions delivered by experienced staff have been shown to be predictive of reliable recovery [8]. Furthermore, patients face additional waiting times between first and second treatment appointments [5]; however, only 1 known study has explored the relationship between these additional waiting times and patient engagement [16].

Furthermore, the NHS Talking Therapies program faces the challenge of patient attrition: only approximately 40% of those referred to the program attend ≥2 treatment sessions [5]. Many patients drop out of the program for unknown reasons; however, the relationship between waiting times and attrition rates cannot be explored using aggregated data. Therefore, tools that enable services to analyze wait durations will enable better research into the relationship between waiting times and patient attrition. Electronic health records (EHRs) capture a rich set of data documenting the details of patient journeys through the Talking Therapies program and patient outcomes. However, the data are complex, and their analysis is not trivial. Treating the care pathway as an operational process allows patient flow and service use to be analyzed using process-centered methods such as process mining.

**Table 1:** The stepped care model for common mental health disorders, revised from the National Institute for Health and Care Excellence guidance (CG123) and the Improving Access to Psychological Therapies manual [10,11].

Step	Examples of conditions	Examples of interventions
1	Presentations of all known and suspected common mental health disorders	Identification, assessment, psychoeducation, active monitoring, and referral for further assessment and interventions
2	Depression (either mild to moderate or persistent subthreshold symptoms), generalized anxiety disorder, panic disorder, and obsessive compulsive disorder	Self-help and guided self-help based on CBT^a^, computerized CBT, psychoeducational groups, behavioral activation, and structured group physical activity program
3	Depression (either moderate or severe, prevention of relapse, or mild to moderate where individuals have not responded to low-intensity interventions), moderate to severe panic disorder, obsessive compulsive disorder with moderate or severe functional impairment, posttraumatic stress disorder, and generalized anxiety disorder, with marked functional impairment or in which individuals have not responded to low-intensity interventions	CBT (individual or group), applied relaxation, mindfulness-based cognitive therapy, interpersonal psychotherapy, behavioral activation, couple therapy, counseling for depression, brief psychodynamic therapy, trauma-focused CBT, EMDR^b^, and graded exercise therapy

^a^CBT: cognitive behavioral therapy.

^b^EMDR: eye movement desensitization and reprocessing.

### Process Mining in Health Care

While traditional business process modeling involves reaching a consensus about model design, process mining is based on the assumption that a process model can be extracted from the information available in systems [17]. Such information can be used to generate “event logs,” the ordered records of events corresponding to the activities encountered by entities that have traveled through a process. An active area of research within process mining is regarding process discovery algorithms, which are often referred to as “process miners” [17]. These algorithms use the information contained within an event log to create a process model that is representative of the behavior captured within the log [18].

In health care, process mining techniques can be used to assess conformance to clinical guidelines and protocols and to assess performance [17]. Frequently asked questions within process mining projects are as follows: “What are the most followed paths and what exceptional paths are followed?” “Are there differences in care paths followed by different patient groups?” “Do we comply with internal and external guidelines?” and “Where are the bottlenecks in the process?” [19].

While process mining has been frequently applied in health care [17,20], in applications such as disease trajectory modeling and clinical pathway analysis [21], research has highlighted that there has been limited uptake within health care organizations, apart from specific research case studies [21,22]. Endeavors are being made toward systematic adoption of process mining in the health care domain through initiatives such as the Process Mining for Healthcare manifesto [21]; however, evidence shows an absence of efforts to integrate process mining tools into systems that record patient data [23].

Recent reviews of process mining in health care [20] and data-driven care pathway mapping from care records [24] have found applications in a number of medical fields, most commonly in oncology; however, neither found evidence of application in the domain of mental health. Outside the field of process mining, the study by Richards et al [12] in 2012 analyzed the delivery of stepped care within mental health services in the United Kingdom; however, there is no evidence of such analysis being developed into a reproducible methodology or tool that could be applied to routinely collected data. Furthermore, a review of the wider literature shows no evidence of the application of process mining to psychological therapy care pathways. The Scopus query “process mining” AND (“psychological therap*” OR “psychological intervention” OR “psychological treatment”) returned only 2 results. Neither of the returned studies explored the care pathway for psychological therapies using process mining. The first study used process mining to explore health care pathways for patients presenting to emergency departments with functional neurological disorders, where onward referral to psychological therapy formed part of the pathway [25]. The second study used data from a psychological therapies service but did so to explore transitions between pre- and posttherapy clinical outcome bands to investigate the impact of appointment attendance on patient outcomes [26].

### Objectives

The application of process mining techniques to NHS Talking Therapies care pathways using EHR data has not been studied; therefore, this study intends to demonstrate the value and utility of doing so. Our study applies process mining to the local care pathways of two NHS Talking Therapies sites and shows how the use, efficiency, and effectiveness of the Talking Therapies care pathway can be explored using a process mining approach. As part of a Knowledge Transfer Partnership project focused on embedding innovation into health care software, our study aims to address the absence of a systematic uptake of process mining by demonstrating how access to analytical tools that allow stakeholders to explore characteristics of their service implementation will enable improved monitoring of system use and better quality analysis into how health systems can affect patient-level outcomes.

## Methods

### Ethics Considerations

The Knowledge Transfer Partnership project titled “Developing innovative, advanced analytical tools to help improve IAPT demand and capacity planning” was approved by the HRA (Health Research Authority) and Health and Care Research Wales (HCRW; integrated research application system project ID 320525), and the University of Bath Psychology Research Ethics Committee (23-031).

### Process Mining

Process mining offers a data-driven approach to analyzing health care processes using the data stored within health information systems in event logs. There are 3 main areas of process mining: process discovery, which involves discovering a process model; conformance, which involves checking an existing process model against an event log; and enhancement, which involves enhancing a model either by repairing incorrect aspects of the model or by extending a model to add a new perspective, including adding extra information such as frequencies, timings, and bottlenecks [18]. In our analysis of Talking Therapies EHR data, we treated the Talking Therapies care pathway as a process, where care pathway stages form the activities in the process, while referred patients (termed “referrals” within IAPT services) are the entities who travel through the process. We demonstrated how process mining can be used for process discovery, evaluation of conformance with system design principles, and performance analysis (enhancement through extension) of the care pathway from referral to discharge.

Throughout our study, the terms “care pathway” and “pathway” are used to describe the whole process of interest, whereas unique sequences of activities are termed “routes” through the care pathway. In the field of process mining, these routes are often referred to as traces or variants.

Research has highlighted the importance of domain expert involvement in process mining projects [21,27,28]. This study is part of a collaborative project with Mayden, the company that provides the “iaptus” digital care record software to NHS Talking Therapies service providers; therefore, expert involvement has been integrated throughout this study. System experts from Mayden work with relevant stakeholders within all Talking Therapies sites using the software to configure each care pathway within the software, ensuring the suitability of the data collected. The specific data for this project were extracted by ETL (extract, transform, and load) experts at Mayden, and data preparation was conducted in consultation with data analysts at Mayden, who are familiar with the data structure and any data quality issues that are universal across sites. The choice of sites for this work was guided by recommendations from service representatives. The approach and results have been reviewed by a user representative from Mayden; furthermore, feedback on this approach was also gathered from Talking Therapies service representatives through user engagement meetings and workshops.

We transformed EHR data into an event log format, manipulated event logs using filtering and stage aggregation, and produced a process map directly from event logs in the form of a directly-follows graph: a descriptive mapping of data to a directed graph of nodes (care pathway stages) and edges (patient flows between stages) in R software (version 4.1.2; R Foundation for Statistical Computing) using the *bupaR* package (version 0.5.2) [29]. Additional R code was developed to extend the process maps with summary statistics and additional formatting, including a bottleneck indicator that uses colors to identify where the edge between the two stages was both highly traveled and had a lengthy median duration. Bright colors are assigned to the edges that fall into the highest percentile when this information is collected into a single metric (the product of the number of patients who moved between the two stages and their median wait duration). A visualization of common routes through the care pathway was built using the *ggplot2* R package (version 3.3.5) [30].

### Data

#### Data Source and Inclusion Criteria

iaptus is the digital care record used by approximately two-thirds of NHS Talking Therapies service providers in England [31]. In this study, we analyzed anonymized EHRs from iaptus relating to the patients referred to two sites between June 1, 2019, and June 1, 2021. These two sites were selected due to their differing size and clinical performance so that the generalizability of the approach could be demonstrated. From the patients referred to the sites during this period, those who consented to their data being processed as part of the IAPT data set, those who were aged ≥18 years at the time of referral, and those who had been discharged by February 8, 2023, were included in study data.

#### Types of Data Used

The NHS target for recovery is that 50% of those who have completed treatment should recover [11]. Recovery is calculated using the notion of clinical “caseness,” which is based on threshold levels of patient reported outcome measures. Patients are considered recovered if they were above the caseness threshold at the beginning of treatment and below the threshold at the end of treatment [11]. Patient reported outcome measures are routinely collected at each session; therefore, patient outcomes for the program have a high degree of data completeness for those who attend ≥2 treatment sessions. Recovery outcome data were available for 94.98% (17,151/18,058) and 96.44% (5034/5220) of the patients who had attended ≥2 sessions at site 1 and site 2, respectively.

Patient geographical data, such as lower super output area (LSOA), was joined with 2019 Office for National Statistics (ONS) data [32] to calculate the proportion of referrals to each site that were assigned to each of the index of multiple deprivation (IMD) deciles. IMD data were available for 99.54% (45,193/45,401) and 99.78% (12,561/12,590) of the patients with referrals to site 1 and site 2, respectively.

The therapist role was available for 71% (375/528) and 82.7% (105/127) of the therapists in the data set for site 1 and site 2, respectively. Role data were mostly missing for the remaining therapists because their role data had been deleted and was no longer available in the system. These therapists had most likely left the service.

Patient appointment records contain information about appointment attendance, which is used to calculate the proportion of patients who completed treatment by attending ≥2 treatment sessions and the proportion of sessions that were not attended. These data were 99.62% (206,564/207,357) and 99.89% (55,415/55,478) complete across site 1 and site 2, respectively. Missing data were removed before calculating the recovery rates, the IMD decile breakdown, the therapist role proportions and missed appointment rates.

Patient movements through the stages of the Talking Therapies care pathway are recorded in the system by service staff as time-stamped events. Each row of data in the event log identifies the referral that the movement relates to, the stage in the pathway that the patient has moved into, and the date and time of the movement. The pathway stages include all aspects of the service’s implementation of the stepped care model, from receipt of referral to assessment, low- (step 2) and high-intensity (step 3) interventions, and the eventual discharge of the patient. Some Talking Therapies services may provide other specialist interventions in addition to step 2 and step 3 treatment; therefore, data were filtered to only include patients with referrals for NHS Talking Therapies treatment.

#### Data Preparation

The event log data needed to undergo data quality assessment and be prepared for analysis. In the analysis, the time stamp of events was used to determine their sequence. However, as the time stamp is recorded manually by staff, there were some issues with the event data, which meant that manually inputted time stamps did not always reflect the actual sequence of events that occurred. Nevertheless, this was imputed from other time stamp data within the movement record, using the time stamps of surrounding events in the sequence, for example, by taking the last observation carried forward. More information on this can be found in Multimedia Appendix 1.

**Figure 1:**
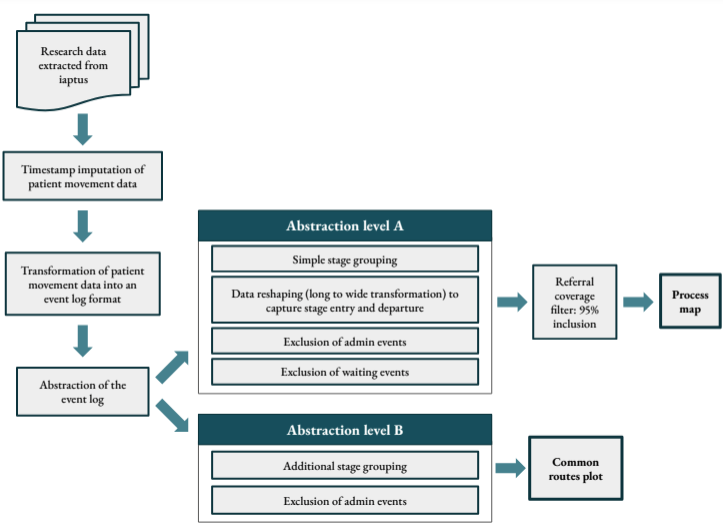
Data preparation steps.

Care pathway configurations can be complex as the pathway is often used within services for patient management and data reporting purposes. In addition, the configuration and the resulting data collected can change over time. For these reasons, a high degree of variation was found in the basic event log; therefore, activities were aggregated and filtered to produce two abstracted event logs for each site that would provide two simplified views of the data set (as shown in Figure 1). These two levels of abstraction were designed in collaboration with data analysts at Mayden.

The abstraction level A involved grouping care pathway stages and excluding events. Grouping care pathway stages included remapping the names of duplicated stages with minor distinctions to appropriate descriptive names; for example, outdated stage names were replaced with newer versions of the name. Other suitable stages were collapsed into overarching subprocesses, whereby consecutive instances of events within the same subprocess are transformed into a single instance of the subprocess. For example, a discharge planning stage was always followed by the final stage representing the discharge of the patient; therefore, these two stages were collapsed into a single “discharged” stage to reduce granularity in the data.

To further reduce the granularity of the data presented in the process map visualizations, events relating to administrative stages, such as moving to a different step intensity or joining a waiting list, were excluded from the event log, as this information was contained implicitly within the data. To produce the process maps, the event log was filtered using a percentage coverage level to include data relating to patients who experienced the most common pathway routes.

For abstraction level B, stage names were remapped in the same way as abstraction level A, and subprocesses were collapsed to a further degree to create larger groups that encompassed more pathway stages. For example, the subprocess “step 3 treatment” included the individual treatment stages at step 3, such as cognitive behavioral therapy (CBT) and counseling. Waiting stage events were not excluded from this version of the event log, as these additional event data proved useful for analyzing distinct pathway routes; however, events relating to other administrative stages were excluded.

## Results

### Data Description

The two sites in our sample differed in size, performance, and referral patient characteristics (Table 2). Site 1 had >3.5 times referrals as site 2. Both sites had similar proportions of referred patients in the lowest IMD deciles; however, site 2 had considerably more referred patients in decile 3 and marginally more patients in deciles 9 and 10, while site 1 had more patients in deciles 5, 7, and 8. On the whole, this suggests that patients referred to site 2 had more variation in deprivation status, whereas site 1 saw a more even distribution across the IMD deciles. Site 2 had a slightly greater proportion of higher-intensity therapists than site 1. While site 1 fell short of the recovery rate target by more than 2%, site 2 exceeded the target by >2%. Site 2 had a higher treatment completion rate (the proportion of referred patients who had ≥2 treatment sessions) and shorter waiting times than site 1 and a higher missed appointment rate. Although site 1 had a lower treatment completion rate, patients with referrals to the site had a marginally higher mean number of treatment sessions than at site 2, suggesting that those who did have treatment had more treatment sessions on average at site 1. The patients with referrals to site 1 were younger, more likely to be female patients, more likely to have an unspecified presenting problem (as opposed to an initial diagnosis), more likely to be self-referred, and less likely to have had a previous referral, in comparison to those with referrals to site 2.

Imputation summary statistics in Table 3 describe the time stamp adjustments for events that exhibited illogical time stamp sequencing. In total, 1.9% (6491/336,637) of the events were adjusted at site 1, with a median adjustment size (the absolute difference between the original value and the imputed value) of 2.3 weeks. In total, 7.2% (8921/123,523) of the events were adjusted at site 2, with a smaller median adjustment size of 0.5 weeks. Across all events (including those with no adjustment), the adjustments averaged very close to 0 at both sites (site 1: mean 0.2, SD 2.3; median 0, IQR 0-0 and site 2: mean 0.1, SD 1.7; median 0, IQR 0-0), suggesting that the impact on the overall results was likely to be minimal. Further information is provided in Multimedia Appendix 1. Furthermore, Table 3 shows the reduction in event log variation from applying abstraction to the log.

**Table 2:** Data summary for the site characteristics, performance measures, and patient referral characteristics present in the data set for discharged patients with referrals received by sites 1 and 2 between June 1, 2019, and June 1, 2021.

				Site 1		Site 2
**Site characteristics**						
	Patient referrals received, N			45,401		12,590
	**Index of multiple deprivation deciles of referrals^a^, n/N (%)**					
		1	4783/45,193 (10.58)		1263/12,561 (10.05)	
		2	5041/45,193 (11.15)		1335/12,561 (10.63)	
		3	4207/45,193 (9.31)		2083/12,561 (16.58)	
		4	4937/45,193 (10.92)		1361/12,561 (10.84)	
		5	4077/45,193 (9.02)		787/12,561 (6.27)	
		6	4091/45,193 (9.05)		1235/12,561 (9.83)	
		7	5502/45,193 (12.17)		1084/12,561 (8.63)	
		8	3980/45,193 (8.81)		682/12,561 (5.43)	
		9	3707/45,193 (8.20)		1194/12,561 (9.51)	
		10	4868/45,193 (10.77)		1537/12,561 (12.24)	
	**Therapist role, n/N (%)**					
		High-intensity therapists	201/375 (53.6)		59/105 (56.2)	
		Low-intensity therapists	174/375 (46.4)		46/105 (43.8)	
**Site performance indicators, n/N (%)**						
	Treatment completion rate (of all patient referrals)			18,058/45,401 (39.77)		5220/12,590 (41.46)
	Recovery rate (of patient referrals who completed treatment)			8212/17,151 (47.88)		2632/5034 (52.28)
	Missed appointment rate (of all scheduled appointments for all patient referrals)			21,757/206,564 (10.53)		6524/55,415 (11.77)
**Summary statistics for patient referrals, mean (SD); median (IQR)**						
		Age (years)	35 (14); 31 (25-42)		38 (15); 35 (26-48)	
		Referral duration (weeks)	23 (23.6);13.9 (4.1-36.9)		18.3 (16.8); 14.7 (2.7-30)	
		Total waiting time duration (weeks)	17.1 (19.2); 8.6 (3.1-23.6)		12.4 (12.8); 7.7 (1-22)	
		Number of treatment sessions (all patient referrals)	3.5 (4.9); 1 (0-6)		3.1 (4.1); 1 (1-5)	
		Number of treatment sessions (patient referrals who completed treatment)	8 (5.1); 7 (5-10)		6.6 (4.4); 6 (3-9)	
**Categorical data for all patient referrals, n/N (%)**						
	**Gender identity**					
		Female patients (including trans women)	30,257/45,401 (66.64)		8179/12,590 (64.96)	
		Male patients (including trans men)	14,934/45,401 (32.89)		4399/12,590 (34.94)	
		Nonbinary patients	192/45,401 (0.42)		6/12,590 (0.05)	
		Unspecified	18/45,401 (0.04)		6/12,590 (0.05)	
	**Presenting problem**					
		Anxiety and stress-related disorders	14,477/45,401 (31.89)		4473/12,590 (35.53)	
		Depression	12,100/45,401 (26.65)		4342/12,590 (34.49)	
		Other mental health problems	1256/45,401 (2.77)		303/12,590 (2.41)	
		Other recorded problems	142/45,401 (0.31)		83/12,590 (0.66)	
		Unspecified	17,426/45,401 (38.38)		3389/12,590 (26.92)	
	**Number of previous National Health Service Talking Therapies program referrals**					
		No previous referrals	38,456/45,401 (84.70)		10,097/12,590 (80.20)	
		1 previous referral	5099/45,401 (11.23)		1874/12,590 (14.88)	
		≥2 previous referrals	1846/45,401 (4.07)		619/12,590 (4.92)	
	**Referral source**					
		Self	42,901/45,401 (94.49)		9853/12,590 (78.26)	
		General practitioner	1432/45,401 (3.15)		867/12,590 (6.89)	
		Other	1068/45,401 (2.35)		1870/12,590 (14.85)	

^a^Where 1 represents the most deprived 10% of lower super output areas.

**Table 3:** Data preparation summary: time stamp imputation and event log abstraction.

Data preparation summary			Site 1			Site 2		
			Values, n	Values, mean (SD)	Values, median (IQR)	Values, n	Values, mean (SD)	Values, median (IQR)
**Imputation summary statistics**								
	**Size of time stamp adjustment (weeks)**							
		Total per referral for all referrals	45,401	1.2 (7.3)	0 (0-0)	12,590	1.5 (7.1)	0 (0-0.1)
		Total per referral for adjusted referrals only	3218	17.4 (21.9)	11 (0.4-25.9)	3489	5.3 (12.8)	1.4 (0.5-3.5)
		Per adjustment for all events	336,637	0.2 (2.3)	0 (0-0)	123,523	0.1 (1.7)	0 (0-0)
		Per adjustment for adjusted events only	6491	8.6 (14.3)	2.3 (0.6-10.2)	8921	2.1 (5.8)	0.5 (0.2-1)
	**Number of time stamp adjustments**							
		Per referral—for all referrals	45,401	0.1 (0.6)	0 (0-0)	12,590	0.7 (1.5)	0 (0-1)
		Per referral— for adjusted referrals only	3218	2 (1.2)	2 (1-3)	3489	2.6 (1.4)	2 (2-3)
**Event log abstraction summary**								
	**Raw event log**							
		Number of unique pathway stages	189	—^a^	—	117	—	—
		Number of unique pathway routes	2454	—	—	1388	—	—
	**Processed event log—abstraction level A**							
		Number of unique pathway stages	26	—	—	23	—	—
		Number of unique pathway routes	388	—	—	268	—	—
	**Processed event log—abstraction level B**							
		Number of unique pathway stages	8	—	—	9	—	—
		Number of unique pathway routes	239	—	—	67	—	—

^a^Not applicable.

### Process Mining

#### Process Mapping

**Figure 2:**
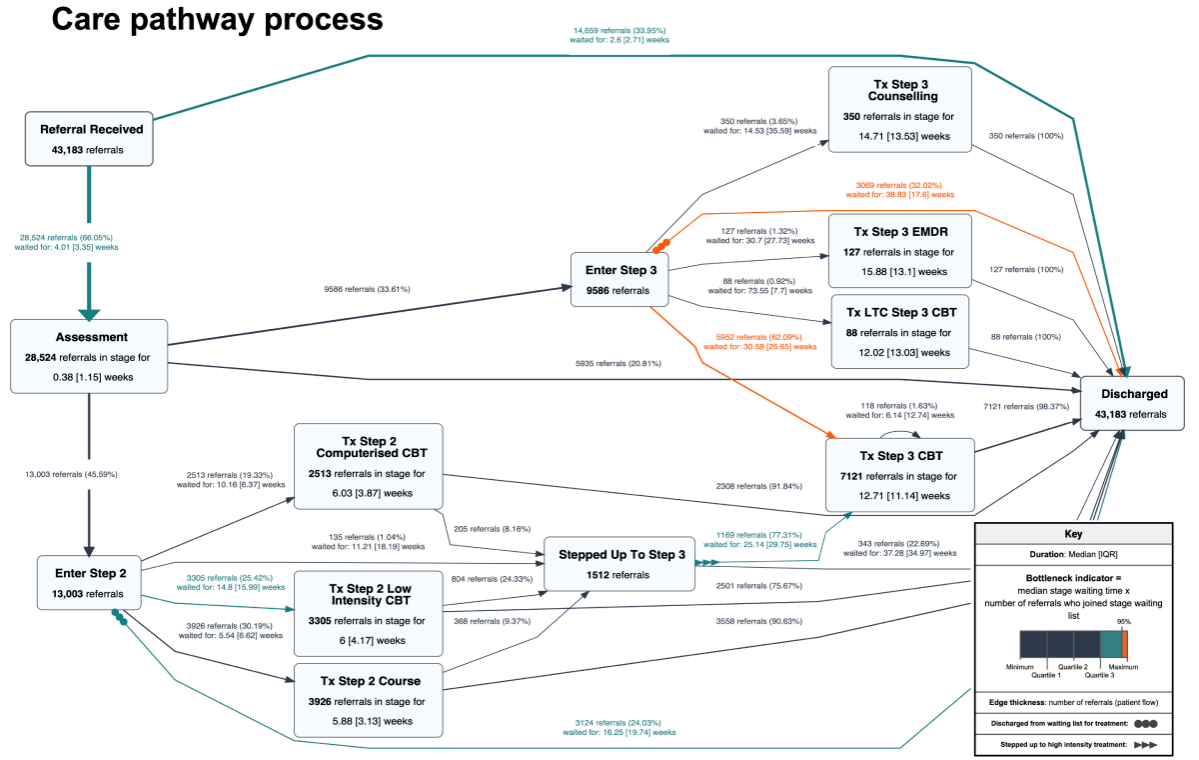
Process map of the care pathway at site 1 using event log A (n=43,183 patient referrals). Referral coverage level=95%. CBT: cognitive behavioral therapy; EMDR: eye movement desensitization and reprocessing; Tx: treatment.

Figure 2 presents a process map with 95% coverage for site 1. The map summarizes the pathway structure, patient flow, and service performance for referred patients who followed common pathway routes. Patients with referrals to the service receive an assessment before being triaged to step 2 (low-intensity treatment) or step 3 (high-intensity treatment). Patients can be “stepped up” from low- to high-intensity treatment (indicated by a triple arrowhead on the edge tail, as shown in the process map key in Figure 2) and can be discharged from any point in the pathway. A process map for site 2 is presented in Figure S1 in Multimedia Appendix 1.

Edge thickness, referral volumes, and branching probabilities demonstrate patient flow. In the process map, one-third (14,659/43,183, 33.95%) of the referred patients were discharged immediately, and a further 20.81% (5935/28,524) of those assessed were discharged after assessment, equating to 13.74% (5935/43,183) of all referrals represented in the map. In total, 27.42% (6193/22,589) of those triaged into steps 2 or 3 were discharged before receiving the intended treatment (3124/13,003, 24.03% of the referrals at step 2 and 3069/9586, 32.02% of the referrals at step 3), equating to 14.34% (6193/43,183) of all the referrals represented in the process map. Flows that involved patients being discharged before receiving the intended treatment are indicated on the map with a set of triple dots on the edge tail (as shown in the process map key in Figure 2). Site 1 had higher attrition rates from waiting lists for treatment than site 2, and at both sites, the attrition rate was higher at step 3. Relatively few patients followed the “step-up” route from low- to high-intensity treatment.

Edge color and duration summary statistics quantify pathway performance for 95.11% (43,183/45,401) of referrals. For example, the initial waiting time for assessment had a median value of 4 weeks, followed by a secondary waiting time for treatment with a median value ranging from 5.5 to 73.6 weeks across treatment types. The duration on each node summarizes the length of treatment (number of weeks) and shows that higher-intensity treatment generally had a longer duration than lower-intensity treatment. The bottleneck indicator highlights the waiting time for step 3 CBT and the waiting time experienced by those who waited for step 3 treatment but were discharged.

#### Common Route Analysis

**Figure 3:**
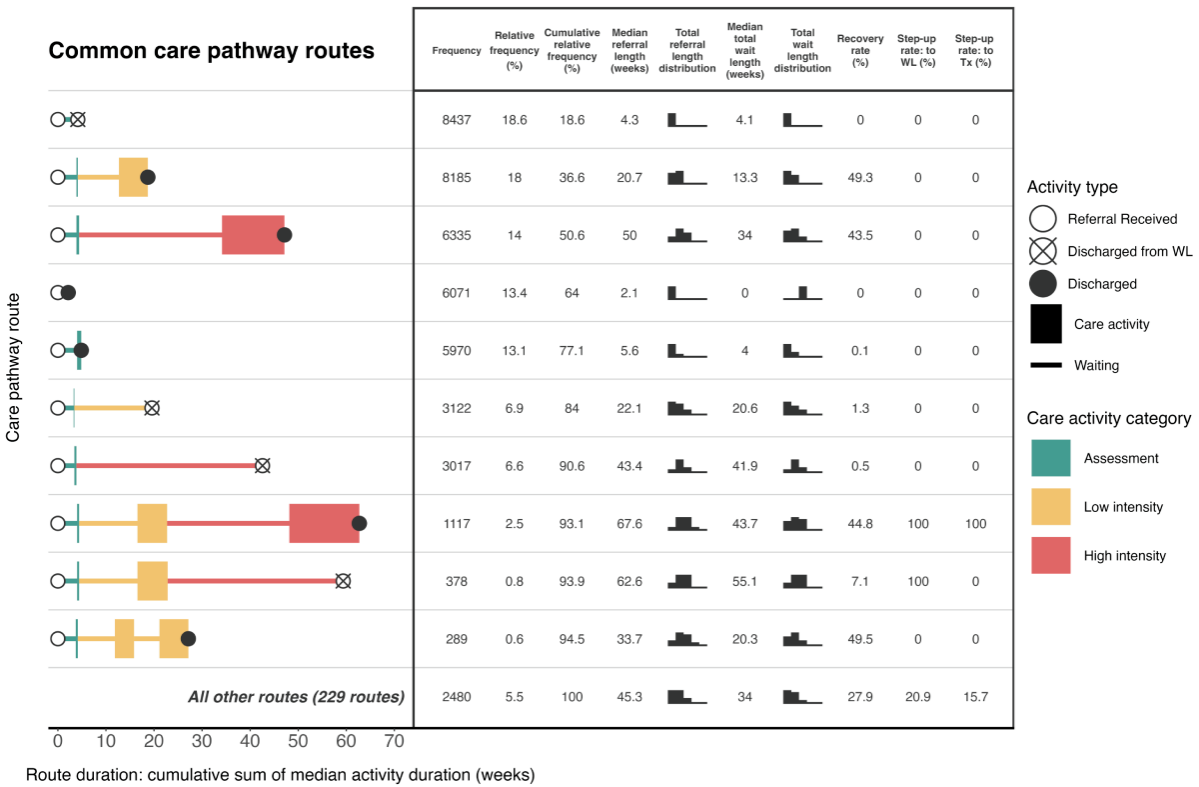
Common route analysis of the care pathway at site 1 using event log B (n=45,401 patient referrals). Coverage level=100%. Top 10 routes only plotted. Tx: treatment; WL: waiting list.

**Figure 4:**
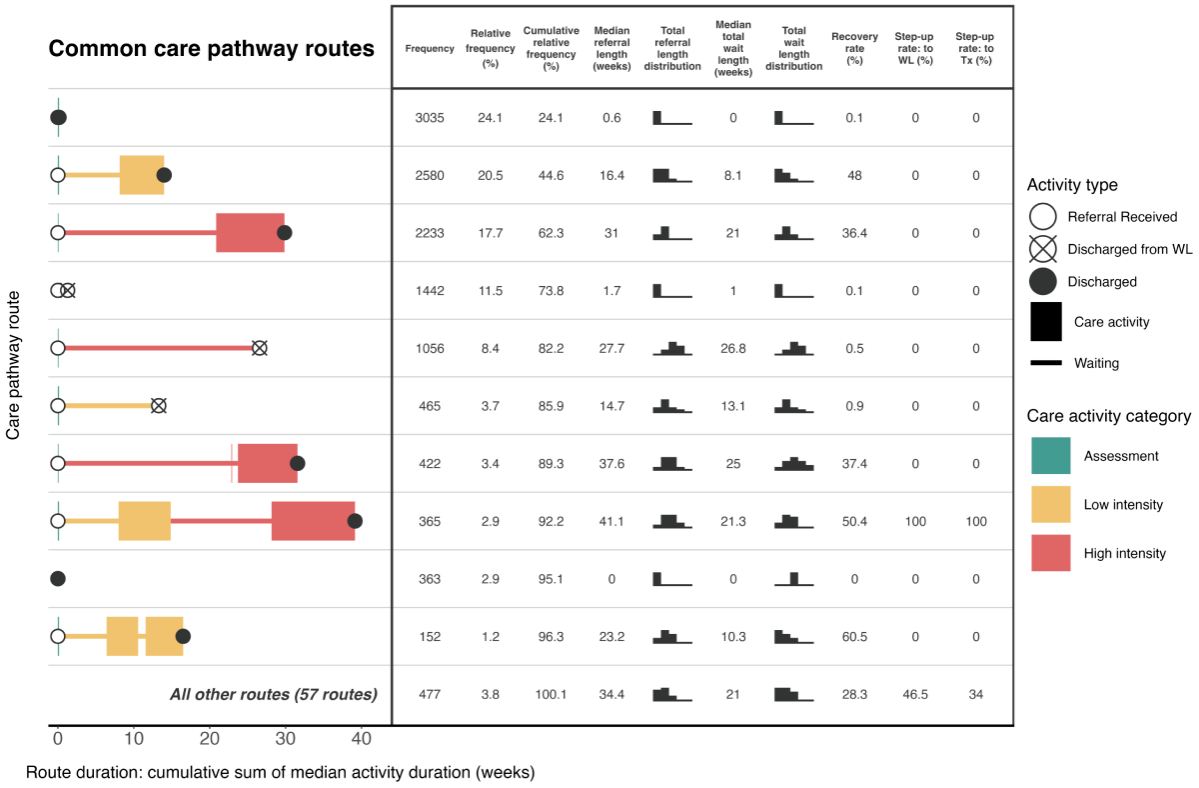
Common route analysis of the care pathway at site 2 using event log B (n=12,590 patient referrals). Coverage level=100%. Top 10 routes only plotted. Tx: treatment; WL: waiting list.

Figure 3 presents the 10 most common care pathway routes at site 1 and the referral outcomes for each route, using event log B. The remaining uncommon routes are summarized under the common routes using the same metrics. The x-axis represents the median duration of activities throughout the course of the referral. Furthermore, a common route analysis is presented for site 2 in Figure 4. The findings from Figure 3 correspond with those from Figure 2 for site 1: three of the 5 most common routes involved no treatment, and routes that involved discharge from waiting lists are also observed. The relative frequencies of common treatment routes show that step 2 treatment alone was more common than step 3 treatment alone, and the comparative infrequency of “stepped care” is evident. Over the common routes at both sites, >5 times as many patients experienced direct access to high-intensity treatment rather than stepped care. In addition, many common routes involved no treatment at site 2. A smaller proportion of patients were discharged from the waiting list for assessment (1442/12,590, 11.45%) in comparison to site 1 (8437/45,401, 18.58%); however, a greater proportion of patients were discharged after being assessed at site 2 (3035/12,590, 24.11%) compared to site 1 (5970/45,401, 13.15%). A total of 1507 of the patients with referrals to site 1 traveled a stepped care pathway route (1117 patients who experienced a common stepped care route + 390 patients who experienced step-up through less common routes), equating to 3.32% (1507/45,401) of all patients referred to the service. At site 2, the equivalent figure was 527 patients (365 patients who experienced a common stepped care route + 162 patients who experienced step-up through less common routes), equating to 4.19% (527/12,590) of all patients referred to the service.

The duration of common pathway routes differed between sites. Longer median waiting times for common assessment and treatment pathway routes were observed for site 1, including the stepped care route. At both sites, the common high-intensity route had a median wait duration of 2.6 times longer than that of the low-intensity route. There was a distinction at both sites between the median wait durations of routes involving discharge from a waiting list, compared to routes where patients completed waiting and commenced treatment. For example, at site 1, the median value of the total wait duration was 13.3 weeks for the step 2 treatment route compared to 20.6 weeks for the attrition alternative. The step 3 treatment route had a total wait duration with a median value of 34 weeks compared to 41.9 weeks for the attrition alternative. At site 2, the equivalent comparisons were 8.1 versus 13.1 weeks at step 2 and 21 versus 26.8 weeks at step 3. This distinction was most severe for the stepped care route at site 1, where those who were stepped up from step 2 but were discharged from the high-intensity waiting list experienced a total wait duration with a median value of 11.4 weeks longer than those who successfully waited for treatment.

## Discussion

In this paper, we demonstrated how process mining techniques, such as process discovery using directly-follows graphs (data-driven process maps), and process enhancement (through extension) using performance and common route analysis can be used to explore “as-is” mental health care pathway use and patient outcomes from a process perspective using routinely collected anonymized EHRs.

### Clinical Guidelines and Outcomes

Our results show how process mining can be used to investigate care system implementation and explore adherence to clinical guidelines and the principles that have informed the design of the care pathway of a service. We presented a data-driven process map of the referrals to a Talking Therapies service over 2 years. The map indicated lower rates of patients being stepped up from low- to high-intensity treatments than might be expected from a “stepped care” system. The common route analysis confirmed that approximately 3% to 4% (site 1: 1507/45,401 and site 2: 527/12,590, respectively) of patients referred to the sites received stepped care.

The stepped care approach is associated with better patient outcomes [8,9], but research has found differences in the implementation of “stepped care” within psychological therapy services [12], for example, a stratified model, where a specific treatment intensity is selected after an initial assessment, versus a progressive model, where low-intensity treatment takes place in the first instance [13]. Therefore, data-driven pathway mapping is a fitting tool to investigate not only the design and implementation decisions of the care system but also the actual use of the system; for example, the National Institute for Health and Care Excellence guidelines instruct that pathways should “allow services to be built around the pathway and not the pathway around the services” [10].

The low stepped care rates presented in this study could be attributed to a number of factors, which could be further explored using process-centered methods. For example, Talking Therapies services are rewarded based on assessment volumes and outcome-based performance [33]; therefore, services can be motivated to process stepped up referrals as separate referrals entirely. Patient-level instances of care, as opposed to referral-level ones, could be used to investigate rereferral pathway routes to explore whether this type of behavior is present in the data. In addition, step-up rates will be impacted by the clinical composition of the referrals received by the service; therefore, by filtering the analysis by individual patient groups, adherence to clinical guidelines and operational principles could be explored further. The relative infrequency of stepped care in comparison to direct access to high-intensity treatment supports the findings of previous research into stepped care implementation [12].

Furthermore, we identified common routes through the Talking Therapies care pathway and presented the clinical performance of these routes in terms of patient recovery. This type of analysis could be used to compare clinical outcomes between pathway routes and could provide a valuable basis for future evaluation of the efficacy of treatment pathways.

### Patient Attrition

Our results established common routes that did not involve treatment. Service managers may wish to monitor routes involving discharge from waiting lists for care. Waiting list attrition is likely to be an undesirable outcome that indicates system inefficiency as well as potentially leading to negative patient outcomes due to untreated symptoms. Analytical tools that incorporate pathway routes can be used to locate patient attrition and monitor patient flows through undesirable routes. For example, although the two sites had similar treatment completion rates of approximately 40% (in line with national rates), route analysis revealed differences in the dominant nontreatment routes between the sites. The distinction between the location of early attrition can help differentiate between that which is patient initiated and that which is attributable to triaging or signposting onward.

Furthermore, we identified attrition later in the course of care, provoking questions regarding the association between system performance and patient attrition. At both sites, the pathway routes involving discharge from a treatment waiting list featured in the most common routes. Moreover, patients who were discharged before receipt of treatment appeared to wait for a longer duration than those who received treatment when comparing the median of the total wait duration of each route. Those discharged before receipt of treatment at site 1 waited 7.3 weeks longer at step 2 and 7.9 weeks longer at step 3 compared to those who received treatment. At site 2, those discharged before receipt of treatment waited 5 weeks longer at step 2 and 5.8 weeks longer at step 3.

In addition, areas of the pathway with longer median waiting times (ie, high-intensity treatment) had higher rates of waiting list attrition. Furthermore, site 2 had shorter median waiting times for treatment and less attrition from the treatment waiting lists than site 1. These initial findings could indicate a relationship between treatment waiting times and patient discharge before treatment commencement. Owing to the limitations of publicly available data, the research literature only seemed to include engagement studies that explored the association between waiting times and appointment nonattendance [15,16] rather than any quantitative study that has explored the relationship between secondary waiting times and patient attrition from the Talking Therapies program. However, our initial results about the relationship between treatment waiting times and patient disengagement are consistent with these studies. This relationship will be explored further in future work, as identifying the factors associated with waiting list attrition could have significant implications for policy.

### Waiting Times and Pathway Bottlenecks

The Talking Therapies program’s waiting times are subject to NHS service standards; however, the initial waiting time for a first appointment is often followed by a secondary waiting period, which, more often than not, is 3 times as long as the initial waiting time [14]. Secondary waiting times are now incorporated into national reporting requirements for Talking Therapies services; however, they are not held to NHS service standards, despite the IAPT manual declaring that they should not be “excessive” [11]. Furthermore, the manual states that the waiting time for high-intensity treatment should not be “substantially longer” than the waiting time for low-intensity treatment and that for those who are stepped up, the waiting time between the low-intensity treatment and high-intensity treatment should “certainly not exceed the waiting time standard for the first intervention” [11].

Accordingly, our analysis provides an overview of system performance in terms of the total wait duration of common pathway routes and further demonstrates patient waiting times throughout the course of care. In addition, the process map bottleneck indicator has been used to highlight areas of the pathway that involve both large patient flows and lengthy median waiting times, indicating pathway stages that might need more urgent attention, and could be used by decision makers to inform capacity allocation or pathway configuration decisions.

Existing studies into the clinical impact of waiting times appear to focus on first waiting times within the pathway. For example, Clark et al [9] used the time between the first referral date and the first treatment appointment as a feature in their predictive models of clinical outcomes. However, as the vast majority of first IAPT assessment appointments are categorized as involving some aspects of treatment [15], it is likely that the first recorded treatment appointment will be the assessment, which will be conducted before the commencement of a course of treatment, leading to potential underestimation of the time taken to enter treatment.

While secondary waiting times are now part of national reporting requirements for Talking Therapies services, these figures are only reported for those who eventually go on to receive a second treatment session; therefore, by definition, they did not drop out before the second treatment session. For this reason, exploration of the impact of these waiting times on patient dropout requires more advanced tools. Analytical tools that allow Talking Therapies services to monitor both initial and secondary waiting times will, therefore, enable better monitoring of excessive waiting times for patients and offer the potential for future research into the relationship between all waiting times and patient outcomes.

### Limitations

Our results are subject to some limitations in relation to the data and preprocessing methods. To create the study data set, referrals in the available data were filtered using both the referral date and the discharge date. The patients with referrals received by the sites within the inclusion window (from June 1, 2019, to June 1, 2021) were excluded from the study sample if they were discharged after the discharge cut-off date (February 8, 2023). This filter could introduce bias toward the end of the referral inclusion window by excluding the patients with referrals with a longer duration; however, the number of patients with referrals received within the referral inclusion window who were not discharged on or before February 8, 2023, was minimal at both sites (292/45,401, 0.64% and 1/12,590, 0.008% of all patients referred to sites 1 and 2, respectively). Therefore, the impact on the overall results is considered to be negligible.

Second, the 2-year period of referral inclusion included the onset of the COVID-19 pandemic, which has been shown to have influenced the rates of access and methods of treatment delivery [5,34]. Some additional analysis has been included in Multimedia Appendix 1 (Figures S2-S8 and Table S1) to evaluate the impact of the pandemic on the results of this study. The volume of referrals to both sites dropped during the initial months of the COVID-19 pandemic and recovered in the months that followed, similar to the early findings presented in the study by Bauer-Staeb et al [34]. There were some differences in performance measures following the onset of the COVID-19 pandemic (Table S1 in Multimedia Appendix 1), such as an increase in recovery rates, a reduction in referral duration and wait duration, a reduction in the missed appointment rate, and changes in the proportion of patients who received ≥2 treatment sessions. These differences can be further explored using the approach presented in this study (Figures S5-S8 in Multimedia Appendix 1) by comparing the frequency and duration of pathway routes across the two time periods. Routes that involved discharge from a waiting list for treatment were less common following the onset of the COVID-19 pandemic. The overall rates of stepped care had some differences following the onset of the COVID-19 pandemic; however, the rates remained low overall, with <6% of all referred patients receiving stepped care across both sites, both before and following the onset of the COVID-19 pandemic.

Third, the imputation techniques applied to illogical event time stamps were based on the assumption that other events’ time stamps could be used as a proxy. Such assumptions were based on our understanding of how iaptus users use the software. In addition, as part of the ongoing development and implementation of this study, elements of data processing that were constructed independently for the two sites, such as the design of the abstraction levels, are being developed into automated criteria that can be applied across all sites. More structured feedback will be gathered from multiple services about the suitability of these assumptions as part of this implementation process during planned future user engagement sessions.

### Clinical Implications

Our study provides a contextual yet structured view of patient flow rather than using isolated metrics to describe pathway use. This study has been driven by the ongoing demand received by Mayden from NHS Talking Therapies services for ways to monitor access to services, explore changes in clinical outcomes, understand patient engagement, and manage service capacity. Ongoing elements of this project include developing the analysis presented in this study into tools that can be used for these purposes across all services that use iaptus, enabling purely data-driven exploration of care pathways from the data that are routinely collected by the services.

Feedback from Talking Therapies service representatives such as service managers, clinical leads, and data analysts through meetings; a webinar with representatives from 33 Talking Therapies services; and user workshops has highlighted that implementing such analysis into routine practice will enable key stakeholders within mental health services to analyze their implementation of the Talking Therapies treatment model, by monitoring actual system use and performance and by exploring how these might impact patient-level outcomes. Further implications proposed by service representatives at these sessions were a better understanding of patient outcomes and engagement, the potential to use this approach to analyze patient movements through the care pathway by their demographic or clinical backgrounds, and the ability to analyze repeated referrals. Service feedback has also suggested that understanding pathway use will support data-driven capacity allocation decisions; therefore, our future research also endeavors to integrate process insights with staff resource information to support such decision-making.

Analysis of patient journeys through pathway routes demonstrates how service users experience the care pathway and is, therefore, naturally patient centered. Integrating this information into an EHR would provide clinicians with an immediate overview of their patients’ previous routes and associated wait times, providing patient-level and service-level insights.

Furthermore, identifying secondary waiting times within the care pathway is a key first step to exploring the relationship between waiting times and engagement outcomes. In addition, our future research aims to model this relationship, enabling services to target improvements to relevant elements of their performance.

## Appendix

Multimedia Appendix 1 Supplemental materials including data preparation details and additional results.

## Abbreviations

CBT: cognitive behavioral therapy
EHR: electronic health record
ETL: extract, transform, and load
HCRW: Health and Care Research Wales
HRA: Health Research Authority
IAPT: Improving Access to Psychological Therapies
IMD: index of multiple deprivation
LSOA: lower super output area
NHS: National Health Service
NICE: National Institute for Health and Care Excellence
ONS: Office for National Statistics

## Data Availability

The data sets analyzed during this study are not publicly available due to the data sharing agreements in place between Mayden (the software provider responsible for iaptus) and the psychological therapies services. This study used anonymized electronic health record data from the digital care record software iaptus.
