# Supplementary material for: Data-Driven Exploration of National Health Service Talking Therapies Care Pathways Using Process Mining: Retrospective Cohort Study
Source: JMIR Ment Health. 2024 May 21;11:e53894. doi: 10.2196/53894 (PMC11150892; doi:10.2196/53894)
Supplement: Multimedia Appendix 1 [file mental_v11i1e53894_app1.pdf]

# SUPPLEMENTAL MATERIAL

## Data driven exploration of NHS Talking Therapies care pathways: an application of process mining to electronic health records.

Elizabeth Yardley [1,2], Alice Davis [2], Chris Eldridge [2], Christos Vasilakis [1].
[1] University of Bath, School of Management, United Kingdom
[2] Mayden, Bath, United Kingdom

## Event log preparation

A data processing script was developed in R to prepare the raw event log data for analysis. The script identified illogical timestamp sequences and imputed each event date that was causing an illogical sequence. Illogical sequences could be confirmed by the order in which these events were added into the system, as recorded by other identifying fields. Three types of illogical sequences were identified, and a different imputation technique was used for each.

Firstly, some events had been backdated. This issue was attributed to care pathway data often being used within services for waiting list management, where waiting lists queues are based on the principle of First In, First Out. Staff could ensure that certain patient referrals would have shorter waiting times by backdating a movement into a waiting list stage. An example of when this might be used is where a patient had already spent some time waiting for a different treatment. This issue was dealt with by imputing the date that the patient was likely to have been moved onto the waiting list for the second time (and similar such scenarios), by using the date of the previous event in the sequence of events as a proxy: effectively taking the Last Observation Carried Forward.

Secondly, some events exhibited a within-day illogical sequence, where the date of the two sequential events matched, but the clock time of the timestamp of the second event preceded the first event. It was determined that this was likely due to iaptus users writing up their caseload into the system after the occurrence of an event. Timestamps of events that exhibited these issues were imputed by carrying forward the last observation, plus one second.

Finally, some events exhibited forward dated timestamp errors. These errors were identified for events with a timestamp that came after the previous event in the event sequence, but did not come before the next event in the sequence, after accounting for cases where the following event in the sequence was backdated as discussed above, which would cause the event in question to appear forward dated. The timestamps for events exhibiting this issue were imputed by using the midpoint of the timestamps of the previous event in the sequence and the following event in the sequence.

## Event log preparation: timestamp imputation results

The median size of an individual adjustment ranged from 2.3 weeks at Site 1, to 0.5 weeks at Site 2. Additionally, on a per referral basis (considering the sum of the adjustments made for each referral), the adjustments were larger at Site 1: 11 weeks, compared to 1.4 at Site 2. The interquartile range (IQR) of the adjustments was also found to be larger at Site 1, both on a per adjustment and a per referral basis. Larger adjustments with higher variability at Site 1 might be attributed to the longer, more varied wait durations experienced at the site. Although the adjustments were larger and more varied at Site 1, far less of the Site 1 sample required adjustments, compared to Site 2, and therefore the overall distribution of adjustments across all referrals was comparable between the two sites. For referrals that did require adjustments, the number of adjustments required, per referral, had a median value of 2. Multiple adjustments were required for consecutive errors within the referral record.

# Additional results

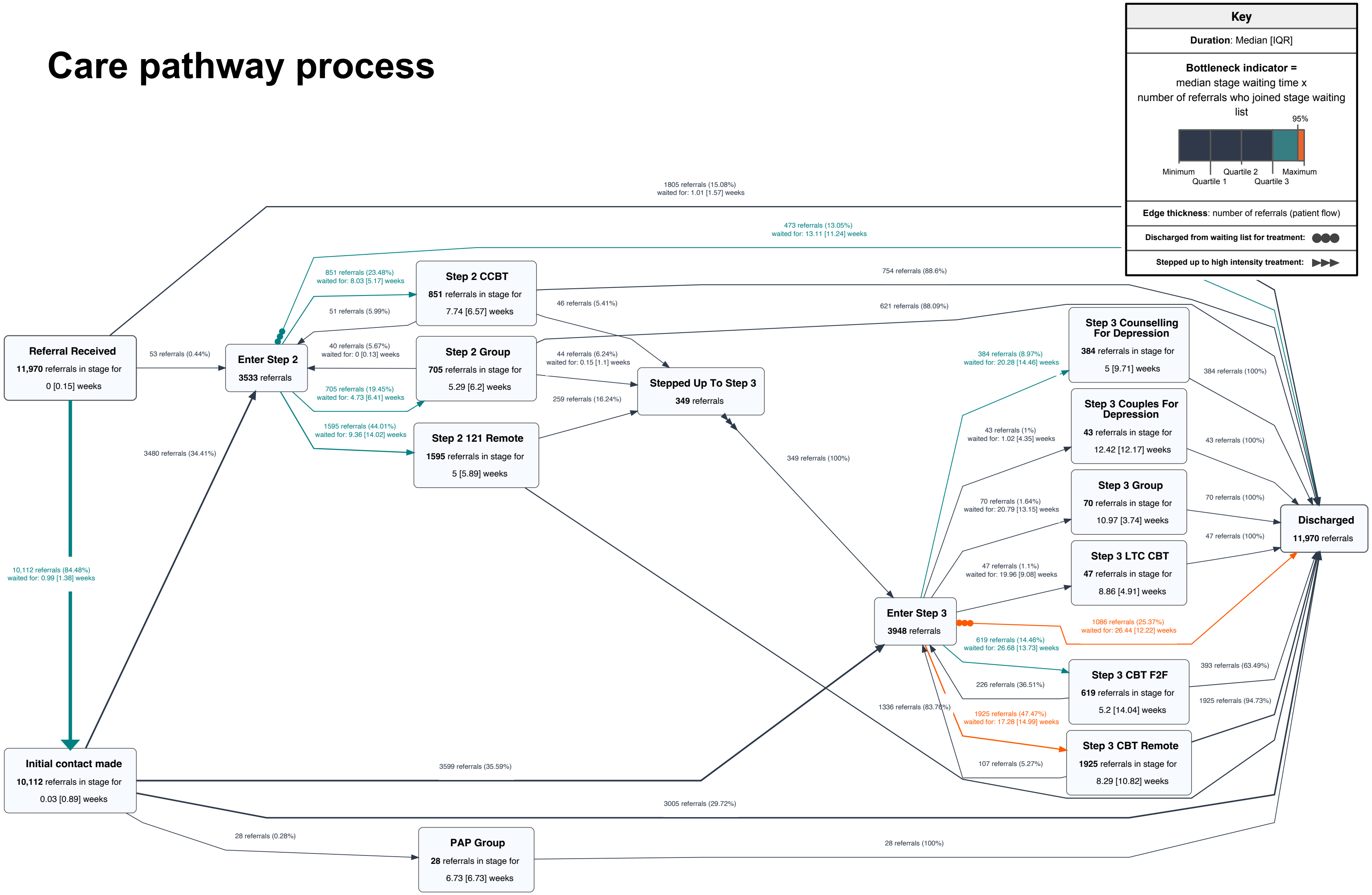


**Figure S1.** Process map of the care pathway at Site 2, using Event Log A. *Referral coverage level = 95%. n(referrals)= 11,970.*

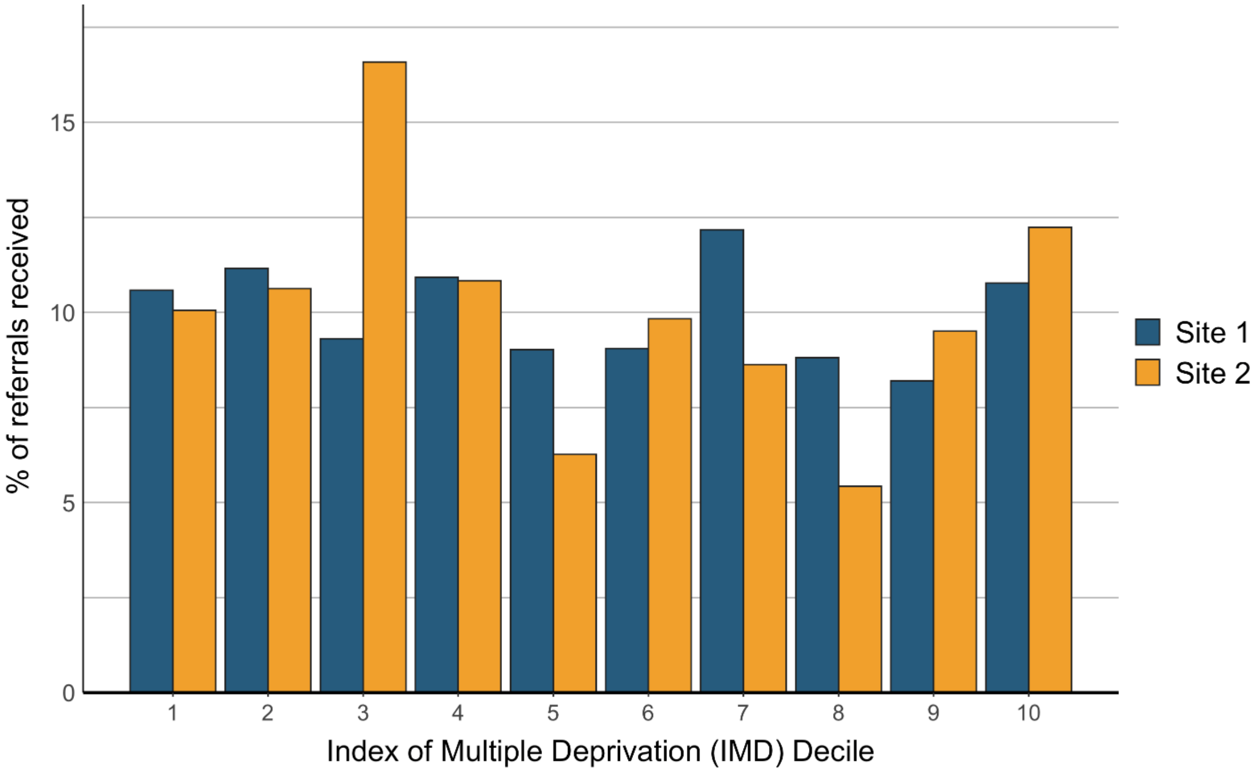


**Figure S2.** Proportion of referrals in each Index of Multiple Deprivation Decile across Sites 1 (*n* = 45, 174) and 2 (*n* = 12,565).

**Table S1.** Data description of discharged referrals received by Sites 1 and 2 between 01-06-2019 and 22-03-2020, inclusive ("pre-pandemic") and those received between 23-03-2020 and 01-06-2021 inclusive ("post-pandemic")

| | Site 1 | Site 2 |
|---|---|---|
| n | 45,401 | 12,590 |
| Pre-pandemic | 15,978 | 5,525 |
| Post-pandemic | 29,423 | 7,065 |
| Performance indicators | % | |
| Treatment completion rate | 39.8 (*n = 45,401*) | 41.5 (*n = 12,590*) |
| Pre-pandemic | 42.0 (*n = 15,978*) | 33.1 (*n = 5,525*) |
| Post-pandemic | 38.5 (*n = 29,423*) | 48.0 (*n = 7,065*) |
| Recovery rate (of referrals that completed treatment) | 47.9 (*n = 17,151*) | 52.3 (*n =5,034*) |
| Pre-pandemic | 45.2 (*n = 6,209*) | 49.7 (*n =1,734*) |
| Post-pandemic | 49.4 (*n =10,942*) | 53.6 (*n = 3,300*) |
| Missed appointment rate | 10.5 (*n = 207,357*) | 11.8 (*n = 55,478*) |
| Pre-pandemic | 13.0 (*n = 78,171*) | 13.7 (*n =19,350*) |
| Post-pandemic | 9.0 (*n =129,186*) | 10.7 (*n = 36,128*) |
| Summary statistics | Mean (SD), Median [IQR] | |
| Age: years | 35 (14), 31 [17] | 38 (15), 35 [22] |
| Pre-pandemic | 36 (14), 33 [19] | 38 (15), 35 [22] |
| Post-pandemic | 34 (14), 31 [17] | 38 (15), 35 [22] |
| Referral duration: weeks | 23 (23.6), 13.9 [32.7] | 18.3 (16.8), 14.7 [27.3] |
| Pre-pandemic | 25.6 (22.7), 21 [34.6] | 21.8 (18.8), 20.1 [33.4] |
| Post-pandemic | 21.6 (24), 10.3 [29.5] | 15.6 (14.6), 12.3 [22.7] |
| Total waiting time duration: weeks | 17.1 (19.2), 8.6 [23.6] | 12.4 (12.8), 7.7 [21] |
| Pre-pandemic | 19.2 (19.1), 13 [27.3] | 16.4 (15.1), 13.3 [27.6] |
| Post-pandemic | 15.9 (19.1), 6.6 [20] | 9.3 (9.5), 5.9 [15.3] |
| Number of treatment sessions (all referrals) | 3.5 (4.9), 1 [6] | 3.1 (4.1), 1 [4] |
| Pre-pandemic | 3.7 (4.9), 1 [6] | 2.4 (3.5), 1 [3] |
| Post-pandemic | 3.4 (4.9), 1 [6] | 3.6 (4.5), 1 [5] |
| Number of treatment sessions (referrals that completed treatment) | 8 (5.1), 7 [5] | 6.6 (4.4), 6 [6] |
| Pre-pandemic | 8 (5), 7 [5] | 6.2 (4), 5 [6] |
| Post-pandemic | 8.1 (5.2), 7 [6] | 6.9 (4.6), 6 [5] |

**Figure S3.** Monthly referrals received by Site 1 between 01-06-2019 01-06-2021 inclusive (complete referrals only).

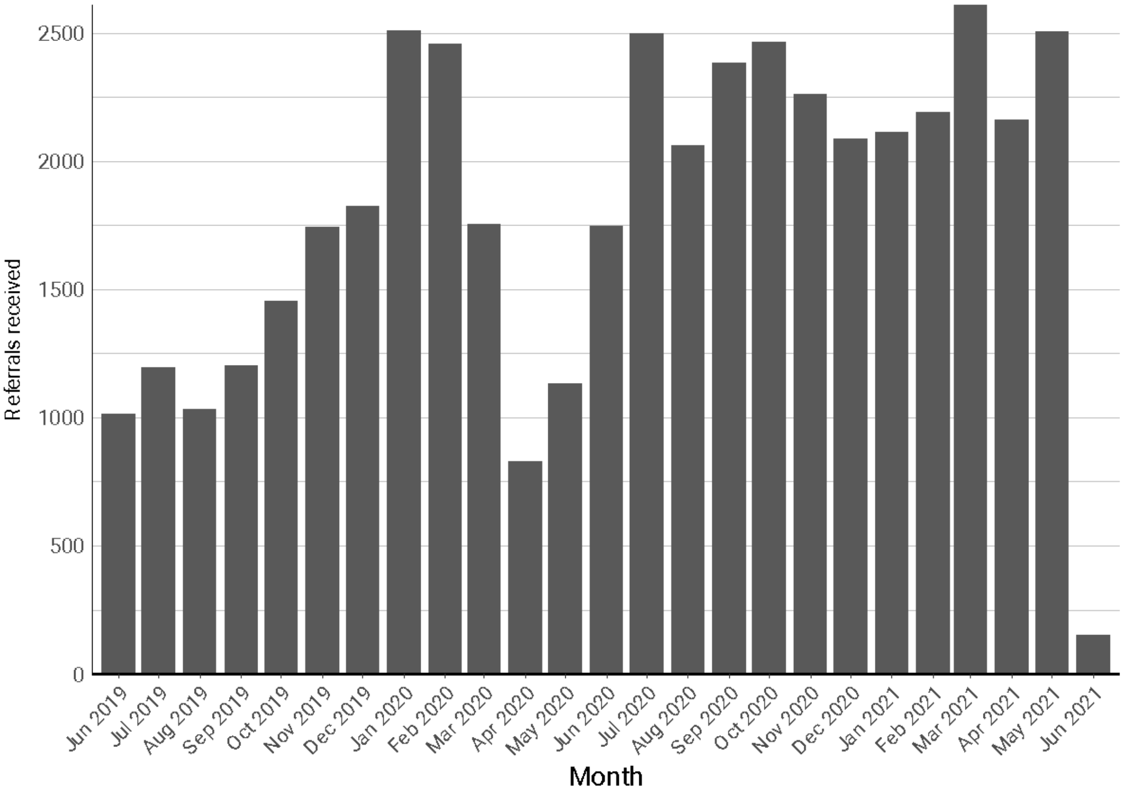


**Figure S4.** Monthly referrals received by Site 2 between 01-06-2019 01-06-2021 inclusive (complete referrals only).

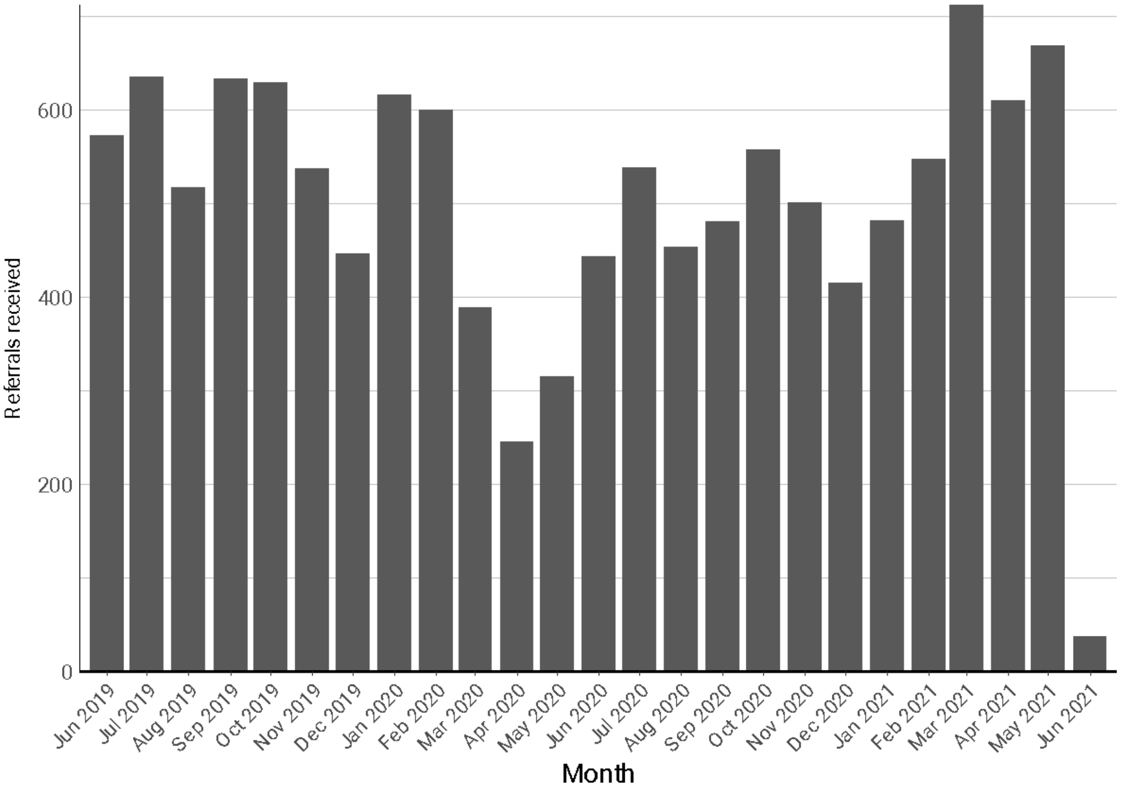

**Figure S5.** Common route analysis of the care pathway at Site 1, using Event Log B, for referrals received between 01-06-2019 and 22-03-2020, inclusive ("pre-pandemic"). *Coverage level = 100%. Top 10 routes plotted only. n(referrals) = 15,978.*

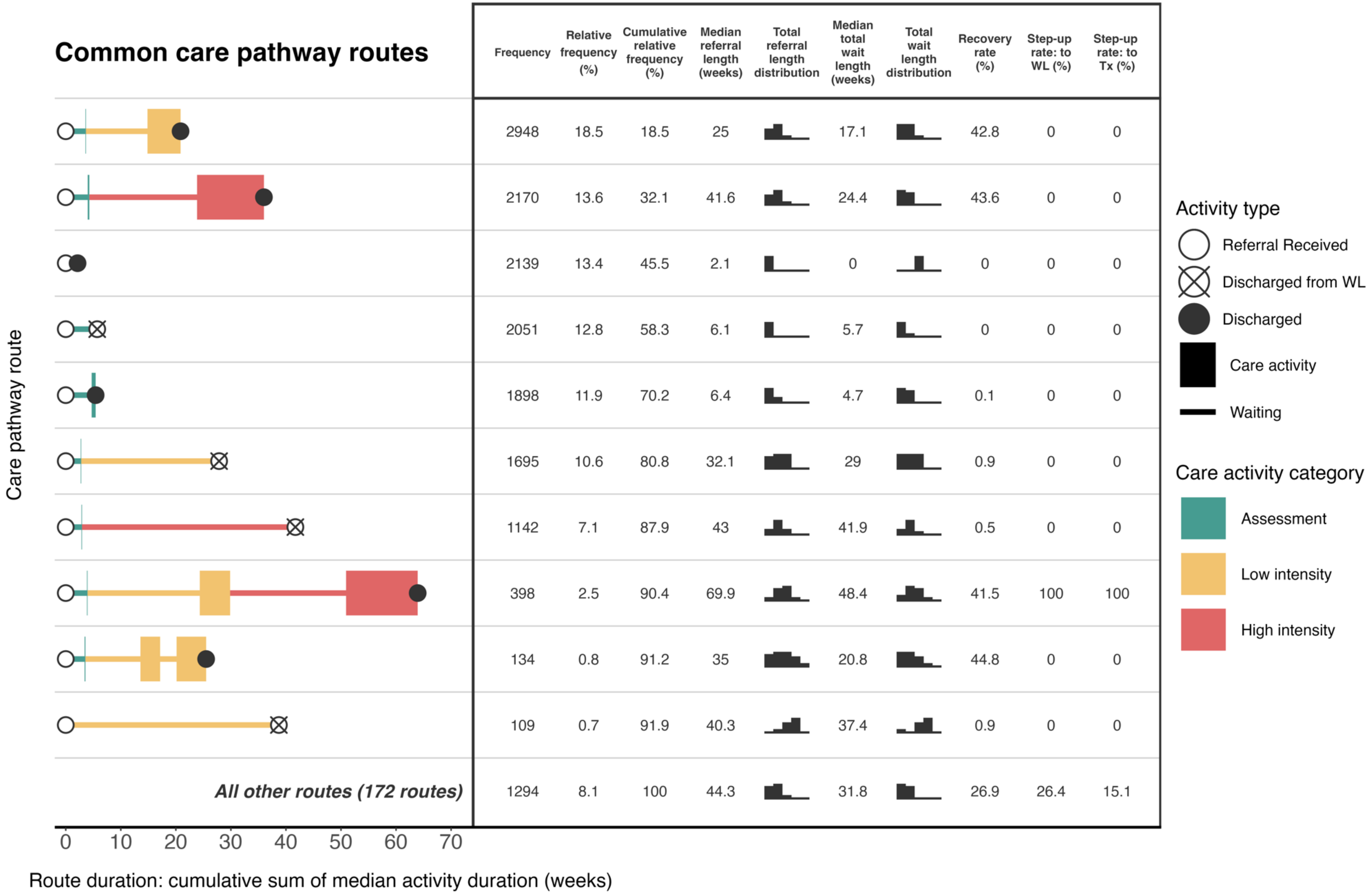


| Frequency | Relative frequency (%) | Cumulative relative frequency (%) | Median referral length (weeks) | Total referral length distribution | Median total wait length (weeks) | Total wait length distribution | Recovery rate (%) | Step-up rate: to WL (%) | Step-up rate: to Tx (%) |
|---|---|---|---|---|---|---|---|---|---|
| 2948 | 18.5 | 18.5 | 25 | | 17.1 | | 42.8 | 0 | 0 |
| 2170 | 13.6 | 32.1 | 41.6 | | 24.4 | | 43.6 | 0 | 0 |
| 2139 | 13.4 | 45.5 | 2.1 | | 0 | | 0 | 0 | 0 |
| 2051 | 12.8 | 58.3 | 6.1 | | 5.7 | | 0 | 0 | 0 |
| 1898 | 11.9 | 70.2 | 6.4 | | 4.7 | | 0.1 | 0 | 0 |
| 1695 | 10.6 | 80.8 | 32.1 | | 29 | | 0.9 | 0 | 0 |
| 1142 | 7.1 | 87.9 | 43 | | 41.9 | | 0.5 | 0 | 0 |
| 398 | 2.5 | 90.4 | 69.9 | | 48.4 | | 41.5 | 100 | 100 |
| 134 | 0.8 | 91.2 | 35 | | 20.8 | | 44.8 | 0 | 0 |
| 109 | 0.7 | 91.9 | 40.3 | | 37.4 | | 0.9 | 0 | 0 |
| 1294 | 8.1 | 100 | 44.3 | | 31.8 | | 26.9 | 26.4 | 15.1 |

**Figure S6.** Common route analysis of the care pathway at Site 1, using Event Log B, for referrals received between 23-03-2020 and 01-06-2021 inclusive ("post-pandemic"). *Coverage level = 100%. Top 10 routes plotted only. n(referrals) = 29,423.*

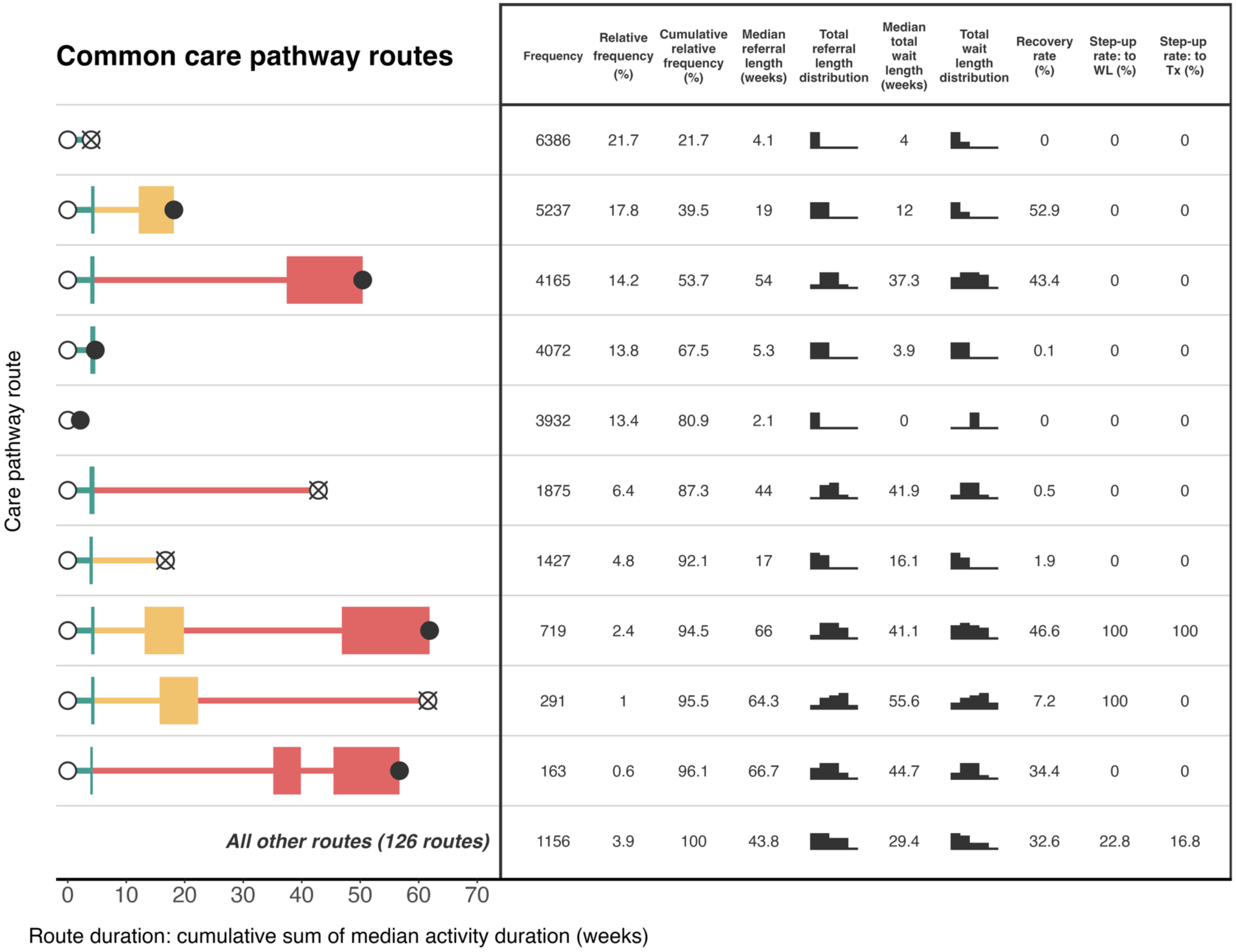


| Frequency | Relative frequency (%) | Cumulative relative frequency (%) | Median referral length (weeks) | Total referral length distribution | Median total wait length (weeks) | Total wait length distribution | Recovery rate (%) | Step-up rate: to WL (%) | Step-up rate: to Tx (%) |
|---|---|---|---|---|---|---|---|---|---|
| 6386 | 21.7 | 21.7 | 4.1 | | 4 | | 0 | 0 | 0 |
| 5237 | 17.8 | 39.5 | 19 | | 12 | | 52.9 | 0 | 0 |
| 4165 | 14.2 | 53.7 | 54 | | 37.3 | | 43.4 | 0 | 0 |
| 4072 | 13.8 | 67.5 | 5.3 | | 3.9 | | 0.1 | 0 | 0 |
| 3932 | 13.4 | 80.9 | 2.1 | | 0 | | 0 | 0 | 0 |
| 1875 | 6.4 | 87.3 | 44 | | 41.9 | | 0.5 | 0 | 0 |
| 1427 | 4.8 | 92.1 | 17 | | 16.1 | | 1.9 | 0 | 0 |
| 719 | 2.4 | 94.5 | 66 | | 41.1 | | 46.6 | 100 | 100 |
| 291 | 1 | 95.5 | 64.3 | | 55.6 | | 7.2 | 100 | 0 |
| 163 | 0.6 | 96.1 | 66.7 | | 44.7 | | 34.4 | 0 | 0 |
| 1156 | 3.9 | 100 | 43.8 | | 29.4 | | 32.6 | 22.8 | 16.8 |

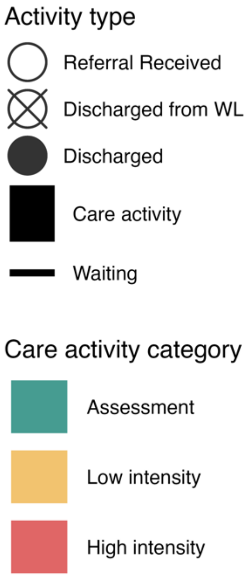

**Figure S7.** Common route analysis of the care pathway at Site 2, using Event Log B, for referrals received between 01-06-2019 and 22-03-2020, inclusive ("pre-pandemic"). *Coverage level = 100%. Top 10 routes plotted only. n(referrals) = 5,525.*

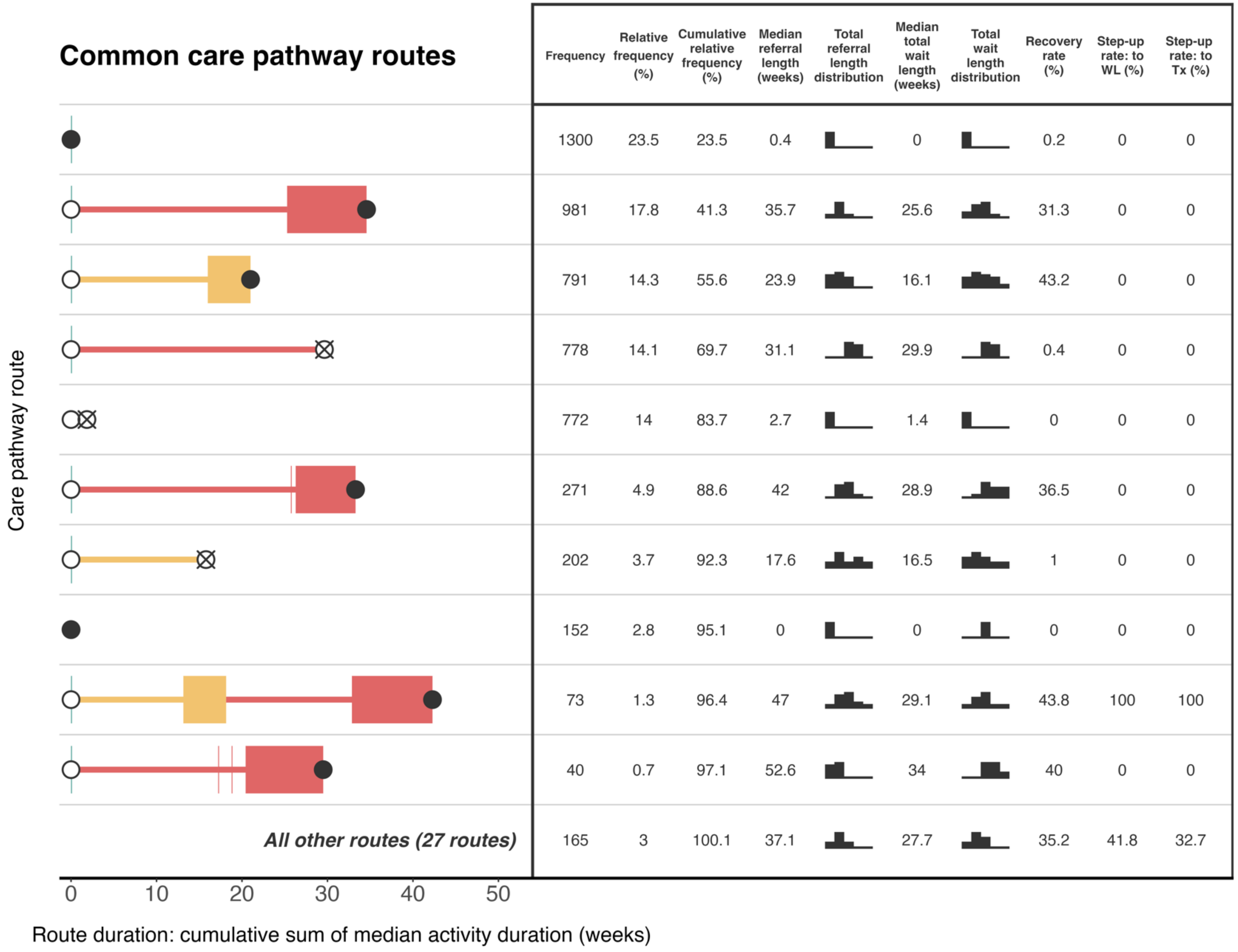


| Frequency | Relative frequency (%) | Cumulative relative frequency (%) | Median referral length (weeks) | Total referral length distribution | Median total wait length (weeks) | Total wait length distribution | Recovery rate (%) | Step-up rate: to WL (%) | Step-up rate: to Tx (%) |
|---|---|---|---|---|---|---|---|---|---|
| 1300 | 23.5 | 23.5 | 0.4 | | 0 | | 0.2 | 0 | 0 |
| 981 | 17.8 | 41.3 | 35.7 | | 25.6 | | 31.3 | 0 | 0 |
| 791 | 14.3 | 55.6 | 23.9 | | 16.1 | | 43.2 | 0 | 0 |
| 778 | 14.1 | 69.7 | 31.1 | | 29.9 | | 0.4 | 0 | 0 |
| 772 | 14 | 83.7 | 2.7 | | 1.4 | | 0 | 0 | 0 |
| 271 | 4.9 | 88.6 | 42 | | 28.9 | | 36.5 | 0 | 0 |
| 202 | 3.7 | 92.3 | 17.6 | | 16.5 | | 1 | 0 | 0 |
| 152 | 2.8 | 95.1 | 0 | | 0 | | 0 | 0 | 0 |
| 73 | 1.3 | 96.4 | 47 | | 29.1 | | 43.8 | 100 | 100 |
| 40 | 0.7 | 97.1 | 52.6 | | 34 | | 40 | 0 | 0 |
| 165 | 3 | 100.1 | 37.1 | | 27.7 | | 35.2 | 41.8 | 32.7 |

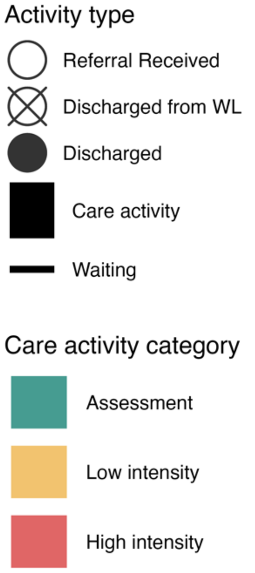

**Figure S8.** Common route analysis of the care pathway at Site 2, using Event Log B, for referrals received between 23-03-2020 and 01-06-2021 inclusive ("post-pandemic"). *Coverage level = 100%. Top 10 routes plotted only. n(referrals) = 7,065.*

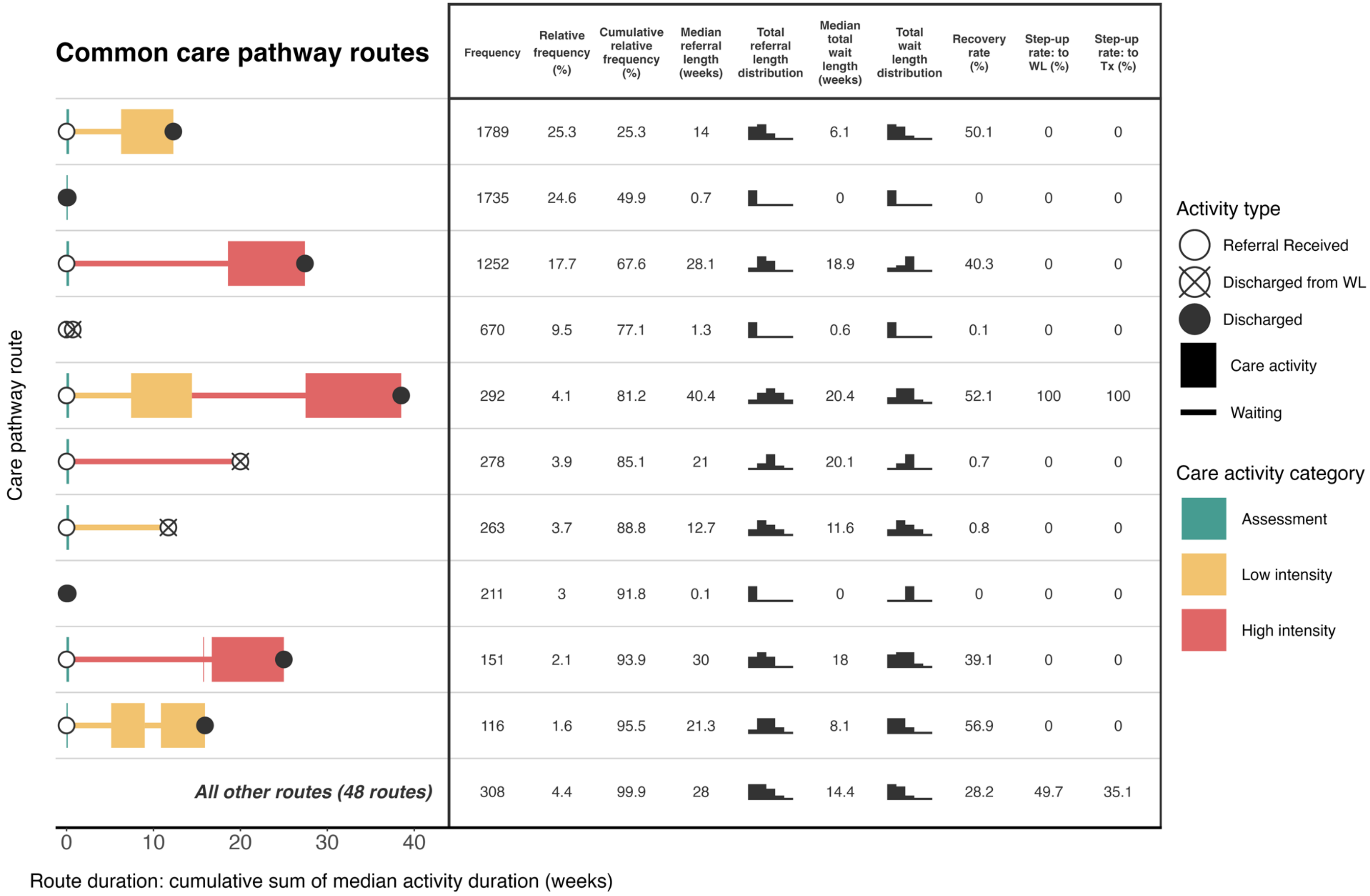


| Frequency | Relative frequency (%) | Cumulative relative frequency (%) | Median referral length (weeks) | Total referral length distribution | Median total wait length (weeks) | Total wait length distribution | Recovery rate (%) | Step-up rate: to WL (%) | Step-up rate: to Tx (%) |
|---|---|---|---|---|---|---|---|---|---|
| 1789 | 25.3 | 25.3 | 14 | | 6.1 | | 50.1 | 0 | 0 |
| 1735 | 24.6 | 49.9 | 0.7 | | 0 | | 0 | 0 | 0 |
| 1252 | 17.7 | 67.6 | 28.1 | | 18.9 | | 40.3 | 0 | 0 |
| 670 | 9.5 | 77.1 | 1.3 | | 0.6 | | 0.1 | 0 | 0 |
| 292 | 4.1 | 81.2 | 40.4 | | 20.4 | | 52.1 | 100 | 100 |
| 278 | 3.9 | 85.1 | 21 | | 20.1 | | 0.7 | 0 | 0 |
| 263 | 3.7 | 88.8 | 12.7 | | 11.6 | | 0.8 | 0 | 0 |
| 211 | 3 | 91.8 | 0.1 | | 0 | | 0 | 0 | 0 |
| 151 | 2.1 | 93.9 | 30 | | 18 | | 39.1 | 0 | 0 |
| 116 | 1.6 | 95.5 | 21.3 | | 8.1 | | 56.9 | 0 | 0 |
| 308 | 4.4 | 99.9 | 28 | | 14.4 | | 28.2 | 49.7 | 35.1 |
